# Antimicrobial Lobophorins from Endophytic Strain *Streptomyces* sp. R6 Obtained from *Azadirachta indica*

**DOI:** 10.3390/molecules30030586

**Published:** 2025-01-27

**Authors:** Xinyuan Chen, Ying Du, Yunlong Ma, Peibin Liu, Yan Chen

**Affiliations:** 1 School of Pharmacy, Liaoning University of Traditional Chinese Medicine, Dalian 116600, China; chenxinyuan967@163.com; 2 Solid Waste and Chemical Management Center, Ministry of Ecology and Environment, Beijing 100029, China; duying92m@163.com; 3 Liaoning Lvyuan Nongfeng Agricultural Technology Service Co., Ltd., Shenyang 110136, China; yunlong_ma@163.com; 4 Institute of Plant Protection, Liaoning Academy of Agricultural Sciences, Shenyang 110161, China; liupeibin5@126.com

**Keywords:** antimicrobial activity, Lobophorins, Polyketides, plant disease, *Streptomyces* sp. R6

## Abstract

Endophytic bacteria are an important source for developing antimicrobial substances. With the aim to find eco-friendly antimicrobial agents from natural sources, *Streptomyces* sp. R6 was isolated from *Azadirachta indica*. After that, a new spirotetronate natural product, lobophorin S (compound **2**), together with lobophorin H8 (compound **1**) and a known macrolide compound divergolide C (compound **3**) were isolated from the cultural solution of strain R6. These compounds mark the first isolation of marine-derived microbial natural products known as lobophorins (LOBs) from endophytic bacteria. The structures of these three compounds were identified by extensive NMR and HRMS analyses. The antimicrobial activities of these three compounds against eight fungal and four bacterial phytopathogens were separately evaluated. Compound **1** demonstrated better antibacterial activity against *Erwinia carotovora*, *Pseudomonas syringae* pv. tomato, and *P. syringae* pv. lachrymans with MIC values of 3.91, 7.81, and 15.63 μg/mL, respectively. Additionally, compounds **1**–**3** all showed antifungal activity against *Botrytis cinerea*, with the MIC values of 1.95, 7.81, and 15.63 μg/mL, respectively. Notably, the *in vivo* antifungal effect of **1** against *B. cinerea* was up to 78.51 ± 3.80% at 1.95 µg/mL, significantly surpassing polyoxin B (70.70 ± 3.81%). These results highlight the potential of lobophorins as promising lead compounds for the development of new, sustainable agents to control plant diseases.

## 1. Introduction

Plant diseases significantly threaten global agricultural production, causing billions of dollars in losses annually [1,2,3]. Despite the widespread use of chemical antimicrobial agents to control these diseases, their overuse has led to several critical issues, such as antimicrobial agent residues, environmental contamination, and the emergence of resistant strains, all of which endanger both human health and ecosystems [1,4,5,6,7]. The increasing resistance of pathogens and the adverse effects of traditional antimicrobial agents underscore the need for sustainable and ecologically friendly alternatives. In this context, natural products and biological antimicrobial agents are gaining recognition as viable substitutes for conventional chemical agents [1,8,9,10].

Plant endophytic actinomycetes are one of the most important groups among endophytes, from which many compounds have been reported [11,12,13] and utilized in agriculture and the pharmaceutical industry [14,15,16]. They are considered one of the best microbial resources for producing bioactive substances, with approximately 80% of secondary metabolites being cultured and isolated from them, among which the genus *Streptomyces* accounts for about 50% [17]. Within this group, it is particularly worth noting that the genus *Streptomyces* is a major source of antimicrobial natural compounds with a variety of chemical structures, such as polyketides (PKs), terpenoids, non-ribosomal peptides (NRPs), and alkaloids, most of which are polyketide compounds [14,16]. A good example is the spirotetronate antibiotic lobophorin (LOBs), which exhibits many types of activities like antimicrobial and anti-tumor activities [18]. LOBs were initially discovered from actinomycetes associated with algae and subsequently re-isolated from the deep-sea-derived *Streptomyces* SCSIO 01,127 [19,20]. LOBs have historically been regarded as marine-derived microbial natural products, and their potential in agricultural production remains unexplored.

Through cultivation and isolation, we obtained an endophytic actinomycete, *Streptomyces* sp. R6, from the leaves of *Azadirachta indica* collected in Thailand [21]. In our search for natural products, the fermentation products of this endophytic strain were extracted, separated, and purified using various phytochemical methods, leading to the isolation of three compounds. The structures of these compounds were accurately elucidated using high-resolution mass spectrometry (HRMS) and nuclear magnetic resonance (NMR) techniques. We employed a modified broth microdilution method to assess the *in vitro* antibacterial effects on four plant-pathogenic bacterial strains—*Pseudomonas syringae* pv. lachrymans, *P. syringae* pv. tomato, *Clavibater michiganensis*, and *Erwinia carotovora*—and eight plant-pathogenic fungal strains, including *Stemphylium solani*, *Fulvia fulva*, *Alternaria solani*, *Botrytis cinerea*, *Pythium aphanidermatum*, *Pyricularia grisea*, *Fusarium oxysporum* (Schl.) f. sp. *cucumerinum* Owen., and *Corynespora cassiicola*. Additionally, we conducted *in vivo* pot experiments to evaluate the effects against the plant-pathogenic fungus *B. cinerea*, in order to provide new clues for the development of antimicrobial molecules.

## 2. Results

### 2.1. Elucidation of Chemical Structures

**Figure 1:**
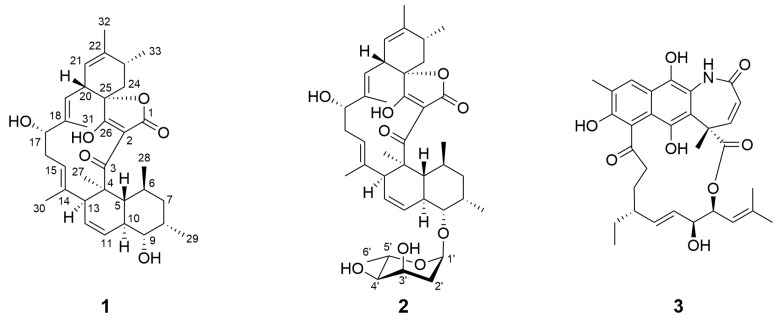
The chemical structures of compounds **1**–**3**.

The extract obtained from the fermentation of strain R6 was fractionated by silica gel column chromatography, Sephadex LH-20 gel chromatography and high-performance liquid chromatography (HPLC), resulting in the isolation of compound **1** (lobophorin H8), compound **2** (lobophorin S, which is characterized as a novel spirotetronate natural product), and a previously identified macrolide compound, compound **3** (divergolide C) (Figure 1) [22].

Compound **1** was obtained as a yellowish-colored solid. Its HRESIMS analysis, in association with ^13^C NMR, and HSQC spectra suggested a molecular formula C_33_H_44_O_6_ with a degree of unsaturation of 12. The ^1^H NMR of compound **1** (Table 1) revealed the presence of five olefinic protons (*δ*_H_ 5.14, br s, H-21; *δ*_H_ 5.26, d, *J* = 10.5 Hz, H-19; *δ*_H_ 5.22, d, *J* = 10.0 Hz, H-15; *δ*_H_ 5.43, m, H-12; *δ*_H_ 6.03, br d, *J* = 10.0 Hz, H-11) and four midfield protons (*δ*_H_ 3.66, dd, *J* = 10.5, 5.3 Hz, H-9; *δ*_H_ 3.53, m, H-13; *δ*_H_ 4.19, br s, H-17; *δ*_H_ 3.58, br d, *J* = 10.5 Hz, H-20). Four singlets and three doublets of methyl (*δ*_H_ 1.28, d, *J* = 6.8, H-33; *δ*_H_ 0.65, d, *J* = 7.0 Hz, H-28; *δ*_H_ 1.04, d, *J* = 6.8, H-29) were also observed in the ^1^H NMR. The ^13^C NMR of compound **1** (Table 1) indicated the presence of 33 carbon atoms. ^13^C NMR and various HSQC spectra suggested that these 33 carbon atoms were distributed as: seven methyl, three methylene, fourteen methane (five sp^2^ and nine sp^3^) and nine quaternary carbon atoms (two carbonyl, one ketone, four olefinic, and two oxygenated carbons). These 33 typically skeletal carbons suggested that compound **1** existed as apentacyclic aglycon featuring atetronate moiety spiro-linked with a cyclohexene ring and belonged to a large class of spirotetronate antibiotics [23]. Moreover, the NMR and mass spectra of compound **1** were consistent with literature reports, and its structure was identified to be lobophorin H8 [24].

**Figure 2:**
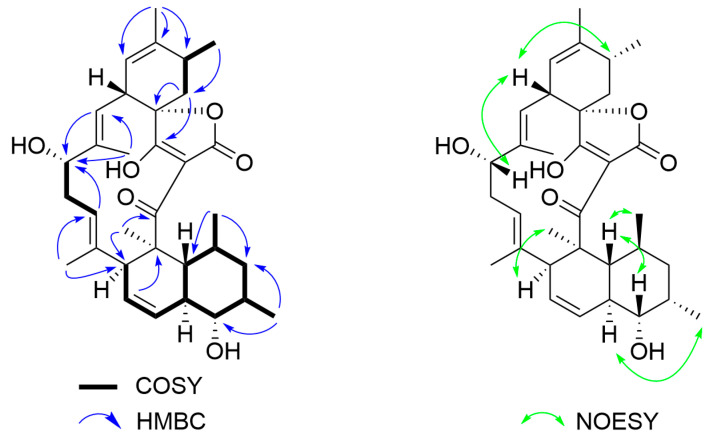
The ^1^H-^1^H COSY, HMBC and NOESY correlations of compound **1**.

The NOESY spectrum of compound **1** provided the following information regarding its relative configuration and interactions: H-5 (*δ* 2.01) to H-9 (*δ* 3.66) and H-5 to H-28 (*δ* 0.65), as shown in Figure 2. This implies that the methyl group at site 28 (H-28) is in close proximity to H-9 and H-5, exhibiting identical orientation. Furthermore, the NOESY correlation was observed between H-10 (*δ* 2.07) and H-29 (*δ* 1.04), demonstrating that H-10 and the 29-CH_3_ group are on the same side of the ring. There are two double bonds in the macrocyclic structure. The NOESY interaction between H-17/H-19 and H-13/H-15 indicate that both pairs are transoriented. Additionally, ∆^11(12)^ was identified as *Z* configuration due to the ^3^*J*(H-C(11), H-C(12) = 10.0 Hz. Thus, the relative structure of compound **1** was finally established and named as lobophorin H8. To the best of our knowledge, this study represents the first report of the isolation of lobophorin H8 from a natural source [20]. Its structure is illustrated in Figure 2.

**Figure 3:**
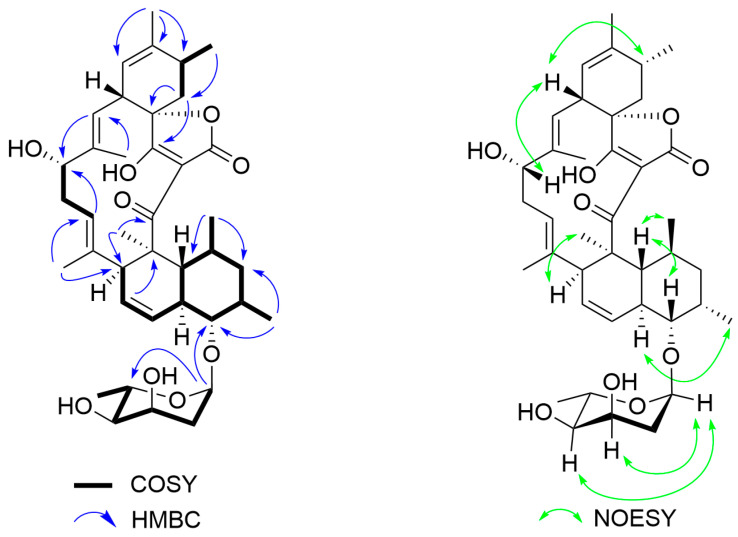
The ^1^H-^1^H COSY, HMBC, and NOESY correlations of compound **2**.

Compound **2** was obtained as a yellowish solid. Its HRESIMS, along with ^13^C NMR, and HSQC spectra revealed a molecular formula C_39_H_54_O_9_ with a degree of unsaturation of 13. The ^1^H NMR spectrum of compound **2** contained five olefinic protons (*δ*_H_ 5.14, br s, H-21; *δ*_H_ 5.27, d, *J* = 10.5 Hz, H-19; *δ*_H_ 5.23, d, *J* = 10.0 Hz, H-15; *δ*_H_ 5.45, m, H-12; *δ*_H_ 5.74, br d, *J* = 10.0 Hz, H-11) (Table 1). The ^13^C NMR and HSQC spectroscopic data (Table 1) of compound **2** indicated 39 carbon resonances, including eight methyl, four methylene, eighteen methane (five sp^2^ and thirteen sp^3^) and nine quaternary carbon atoms (two carbonyl, one ketone, four olefinic and two oxygenated). From these characteristic signals, it can be deduced that compound **2** belonged to a large class of spirotetronate antibiotics and was highly similar in structure to compound **1** according to the NMR data (Table 1). The only difference between compounds **2** and **1** is a substitution of glycosidic moiety in **2**, which was found according to six carbon signals (including one methyl at *δ*_C_ 17.7, one methylene at *δ*_C_ 34.9, and four oxygenated methine at *δ*_C_ 99.6, 72.6, 67.1, and 65.3) in the ^13^C NMR spectra of **2**. The key HMBC correlations (Figure 3) of H-1′ with C-9 (*δ*_C_ 85.6) and C-5′ (*δ*_C_ 65.3) confirmed that a digitoxose unit was established located at C-9 in **2**, which was further verified by the correlations of H-11 (*δ*_H_ 5.74) with H-9 (*δ*_H_ 3.57) from ^1^H-^1^H COSY spectrum (Figure 3).

The relative configuration of compound **2** was determined through NOESY experiments. In the NOESY spectrum (Figure 2), the correlations of H-17 with H-23 suggested that 17-OH in **2** might be *α*-oriented. An *α*-orientation for 9-*O-*L-digitoxose was deduced from the NOESY correlations of H-1′ with H-3′/H-4′. Therefore, the structure of compound **2** was established and designated as lobophorin S.

### 2.2. In Vitro Antimicrobial Activity Assay

The 96-well plate method was employed to evaluate the *in vitro* inhibitory activities of the three compounds against four bacterial and eight fungal plant pathogens [25,26,27]. The test results of the inhibitory activities were presented in terms of their Minimum Inhibitory Concentration (MIC). As shown in Table 2, compounds **1**–**3** exhibited moderate to good antimicrobial activity and exhibited a relatively broad antimicrobial spectrum against various pathogenic bacteria. Compound **1** displayed significant antibacterial effect against all tested pathogenic bacteria, particularly against *Erwinia carotovora* with an MIC value of 3.91 μg/mL, which was much lower than that of streptomycin (31.25 μg/mL). Compound **3** was also effective against *E. carotovora* with an MIC value of 31.25 μg/mL. Additionally, compound **2** demonstrated significant growth inhibition for *Pseudomonas syringae* pv. tomato, with an MIC value of 15.63 μg/mL. All three compounds exhibited antifungal activity against *Botrytis cinerea* with MIC values of 1.95, 7.81, and 15.63 μg/mL, respectively, which were similar to the positive control polyoxin B (3.91 μg/mL) but higher than that of carbendazim (31.25 μg/mL) and amphotericin B (125 μg/mL). Additionally, compound **1** and **3** showed good inhibitory activity against *Fulvia fulva*, with an MIC value of 31.25 μg/mL (Table 3).

In the *in vitro* antimicrobial activity assay, the three compounds exhibited varying degrees of activity against a range of plant pathogens. This variability can be attributed to two primary factors: firstly, the distinct species of each strain, which possess varying levels of drug susceptibility and resistance; secondly, the differing mechanisms of action and targets of the compounds themselves. These factors, among others, contribute to the significant differences in activity observed across different strains. Furthermore, even the positive controls demonstrated considerable variation in their inhibitory effects.

### 2.3. Antifungal Activity Against Botrytis Cinerea In Vivo

We further investigated the inhibitory activity of compound **1** against gray mold in tomatoes using pot experiments [28,29]. The spray method was selected to inoculate uniformly growing tomato seedlings, and then, compound **1** was applied to the inoculated tomato plants at five gradient concentrations. The experimental results show that as the concentration of compound **1** increased, a dose-dependent control effect on the gray mold pathogen was observed (Table 4). Notably, at a concentration of 7.81 µg/mL, the *in vivo* control effect on *B. cinerea* reached as high as 85.44 ± 3.29%. When the concentration was reduced to 3.91 µg/mL and 1.95 µg/mL, the control effects were 80.73 ± 2.42% and 78.51 ± 3.80%, respectively. At the 0.05 level, there was no statistically significant difference between the two treatments. At a concentration of 1.95 µg/mL, the *in vivo* control effect of polyoxin B on *B. cinerea* was 70.70 ± 3.81%, which was significantly lower than that of compound **1**.

## 3. Discussion

Lobophorins (LOBs) are a subset of spirotetronate antibiotics with over 60 naturally occurring variants [30] that share a similar skeletal structure with kijanimicin [31]. LOBs have achieved certain therapeutic value in clinical aspects [31,32]. Historically reported LOBs have been isolated from marine microorganism algae and streptomycetes [19,20,33,34,35,36,37,38]. Interestingly, three compounds have been isolated and identified from the plant endophytic actinomycete strain R6, including one new and one known LOB, marking a departure from the traditional marine sources. Therefore, our research adds to a chemical diversity in LOBs.

Crop diseases caused by phytopathogenic fungi and bacteria represent a significant threat to agricultural production, leading to substantial losses in crop yield and quality annually [1,2,3,39,40]. Among these, *B. cinerea*, a serious plant pathogen [41], has been classified as a high-risk of resistance to many known fungicides [42,43]. The excessive use of high-concentration fungicides can result in the formation of toxic residues, increased drug resistance [44], and also poses environmental hazards [45,46]. The growing resistance of plant pathogens to conventional treatments and the environmental concerns associated with chemical fungicides have driven people to seek novel and effective solutions. LOBs exhibit a wide range of biological activities, and their development and utilization have significant medical value [47]. As of now, LOBs that have been isolated exhibit antibacterial activity against a variety of bacteria. LOBs F, H, and K demonstrate antimicrobial effects against *Staphylococcus aureus* [20,35,37]. LOB F also shows activity against *Enterococcus faecalis* [20] and *Bacillus subtilis* [35]. LOBs G, A, and B are effective against *B. Calmette-Guerin* (BCG), *Mycobacterium tuberculosis* H37Rv, and *B. subtilis* [41], and are also effective against *Mycobacterium smegmatis* MC^2^ 155 [48]. LOB L shows significant activity against *Micrococcus luteus* and *B. thuringiensis* [38]. LOBs H, I [35] and H8 display activity against *B. subtilis*, with LOB H8 also showing activity against *M. smegmatis* MC^2^ 155 [48]. To date, the isolated LOBs have demonstrated antibacterial activity against a multitude of bacterial strains. Regarding antifungal activity, specific reports on LOBs are notably absent. In this study, both LOBs showed a broad spectrum of inhibitory effects against plant pathogenic bacteria and fungi. *In vitro* tests demonstrated that LOB H8 effectively inhibited three bacterial growths. Furthermore, *in vivo* tests confirmed the bioactivity of the compound against *B. cinerea*. The isolation of LOBs from endophytic plant actinomycetes not only diversifies the structural landscape of polyketide compounds but also highlights the potential of natural products as a source for developing new and efficient active ingredients to combat plant diseases [15,49]. We will continue to explore the mechanisms of the antimicrobial activity of these compounds, providing a foundation for their application in agricultural production.

## 4. Materials and Methods

### 4.1. Isolation of Streptomyces sp. R6

*Streptomyces* sp. R6 was isolated from the neem plant by the standard technique and tissue homogenate technique [50,51], and it was identified by phylogenetic analysis and comparison of its 16S rDNA sequence with the sequences available in the EzTaxon database. It was found that the strain R6 exhibits the highest similarity with *Streptomyces pactum* NBRC 13433T and *Streptomyces olivaceus* NRRL B-3009T, with homologies of 99.09%. Consequently, the strain was named *Streptomyces* sp. R6 (Genbank accession no. MF375233). Strain R6 was preserved in the Laboratory of Pesticide Science, College of Plant Protection at Shenyang Agricultural University, China.

### 4.2. Production and Extraction of Antimicrobial Compounds

The production and extraction of *Streptomyces* sp. R6 were conducted in accordance with the methods described below [52]. Fermentation of strain R6 was carried out using an optimized formula of F medium, i.e., glucose, 10.0 g; soluble starch, 20.0 g; peptone, 5.0 g; NaCl 4.0 g; yeast extract, 5.0 g; MgSO_4_·7H_2_O, 0.5 g; K_2_HPO_4_, 0.5 g and CaCO_3_, 2.0 g per liter of medium with pH of 8.0. The starter (OD_600_ = 0.9) of *Streptomyces* sp. R6 was placed in a 250 mL flask, which was then incubated at 28 °C for 48 h in a rotary shaker (Zhichu, Shanghai, China) at 180 rpm. For mass fermentation, a total of 90 L of the medium was used, and 400 mL of medium was inoculated into each 2 L Erlenmeyer flask with 20 mL of starter culture. All the flasks were incubated under identical conditions for 7 days. Mycelia were removed by centrifugation at 6000 rpm and 4 °C for 30 min. The aliquot broth was shaken with 4% Amberlite XAD-16 (ROHM and HAAS, Philadelphia, PA, USA) resin for 4 h at 25 °C. The resins were harvested by filtration through a double layer of muslin cloth and dried at 28 °C. The fully dried resins were transferred to a separation funnel and extracted with methanol (500 mL). The methanol extract was concentrated using a rotary evaporator (Buchi, Switzerland) under reduced pressure. This MeOH extract was concentrated using a rotary evaporator at 28 °C to yield 11.58 g of crude solid residue.

### 4.3. Purification

The purification of the fermentation products was conducted following the methods outlined below [25]. The MeOH extract was divided into six fractions (fractions C1–C6) using various ratios of CH_2_Cl_2_−MeOH (100:0, 100:2, 100:4, 100:8, 100:16, and 0:100, 2 L each) as the mobile phase with a silica gel column (100–200 mesh, 350 mm × 25 mm i.d.). The compound **1** (48.2 mg, about 98% purity) and a mixture were obtained from fraction C1, which was collected using CH_2_Cl_2_-MeOH (100:1.6) as the mobile phase in another silica gel column (200–300 mesh, 350 mm × 15 mm i.d.). The compound **2** (t_R_ = 35.6 min, 24.4 mg, about 96% purity) was isolated from the mixture by reverse-phase semipreparative HPLC (Agilent, Santa Clara, CA, USA), equipped with a 250 mm × 9.4 mm, 5 μm, ZORBAX Eclipse XDB C_18_ column (Agilent, Santa Clara, CA, USA) eluted with 85% CH_3_OH + 0.1% HCOOH as mobile phase at a flow rate of 3 mL/min for 40 min and UV detector at 210 nm. Fraction C2 which was collected using CH_2_Cl_2_-MeOH (100:2) as the mobile phase was subsequently chromatographed on a Sephadex LH-20 (GE Healthcare, Uppsala, Sweden) column (500 mm × 20 mm i.d.) using CH_2_Cl_2_-CH_3_OH (1:1) as mobile phase to obtain fractions C2a and C2b. Further purification C2b was performed by reverse-phase semipreparative HPLC (flow rate: 3 mL/min for 38 min, UV detection: 210 nm) (Agilent, Santa Clara, CA, USA), eluted with 45% CH_3_CN + 0.1% HCOOH to obtain 81.6 mg, about 97% purity, of compound **3** (t_R_ = 31.6 min).

For structural elucidation, NMR spectra were recorded using an NMR spectrometer (Avance-600) (Bruker, Karlsruhe, Germany) operated at room temperature. The instrument was calibrated with carbon signals of CDCl_3_ (*δ*_C_ 77.0 and *δ*_H_ 7.26) and residual proton signals. AP-300 polarimeter (Atago, Tokyo, Japan) was utilized to measure optical rotations. A 6500 series quadrupole-time-of-flight (Q-TOF) mass spectrometer (Agilent, Santa Clara, CA, USA) was used to record high-resolution electrospray ionization mass spectrometry (HRESIMS) data.

### 4.4. In Vitro Antimicrobial Assay

A modified broth microdilution method [25,26,27] was employed to evaluate the *in vitro* antimicrobial activities of purified compounds against four bacterial strains (*Pseudomonas syringae* pv. lachrymans, *P. syringae* pv. tomato, *Clavibater michiganensis*, *Erwinia carotovora*) and eight fungal strains (*Stemphylium solani*, *Fulvia fulva*, *Alternaria solani*, *Botrytis cinerea*, *Pythium aphanidermatum*, *Pyricularia grisea*, *Fusarium oxysporum* (Schl.) f. sp. *cucumerinum* Owen., *Corynespora cassiicola*) in 96-wells microtiter plates. Bacterial and fungal strains were obtained from the Laboratory of Plant Science, College of Plant Protection, Shenyang Agricultural University, China.

Bacteria and fungi were cultured in a nutrient broth (NB) and potato dextrose broth (PDB), respectively, and subsequently prepared as mixed suspensions [25,26,27]. The medium volume per well was 100 μL. These fungi and bacteria were inoculated in Roswell Park Memorial Institute 1640 medium and Mueller–Hinton broth medium. Solutions of three purified compounds and positive controls (amphotericin B, streptomycin, carbendazim, and polyoxin B) were prepared in Dimethyl sulfoxide (DMSO) with a two-fold step dilution ranging from 0.98 to 500 µg/mL. A 1% DMSO was used as the negative control, representing the highest concentration used in the assay. A total of 10 µL (10^6^ cfu/mL) of bacterial and fungal pathogen suspensions were added to each well. After mixing, the bacterial cultures were incubated at 24 °C for 24 h and the fungi at 30 °C for 48 h. The experiment was repeated three times. A microplate reader (Molecular Devices, San Jose, CA, USA) was used to record the optical density (OD) of each well at 600 nm. The lowest concentration to show 100% inhibition of the growth of the pathogen was defined as the minimum inhibitory concentration (MIC). The percentage inhibition of each dilution was calculated using the following formula:

Inhibition (%) = 100 × [(1 − Treatment OD)/negative control OD]

### 4.5. In Vivo Antifungal Activity Against Botrytis Cinerea by Greenhouse Pot Experiments

The *in vivo* activity of compound **1** against *B. cinerea* was tested under greenhouse conditions, with references to and improvements upon the tomato pot experiment [28,29]. Disinfected tomato seeds were treated with warm water to promote germination, and seeds with good germination were grown in plastic pots containing a 1:1 (*v*/*v*) mixture of vermiculite and peat. When the tomato plants reached the 6-leaf stage, they were inoculated with a mycelial suspension of *B. cinerea* (5 × 10^8^ cfu/mL), applying 3 mL per plant. After 24 h, the plants were treated with Compound 1 at various concentrations (0.49, 0.98, 1.95, 3.91, and 7.81 µg/mL), applying 1 mL per plant. Polyoxin B was used as the positive control and distilled water as the negative control. After treatment, the tomato plants were placed in a sealed space at 20 °C and 85% relative humidity (RH), and a light/dark cycle of 16/8 h for 7 d. All treatments were replicated three times. Polyoxin B was established as the positive control, while distilled water served as the negative control. Antifungal activity data were recorded when the negative control (CK) showed significant symptoms of disease. The disease index (DI) and control effect (I) were calculated as follows:

DI = [∑ (number of diseased leaves at each grade × level of disease)/ (total number of leaves × 9)] × 100

I (%) = [(CK − T)/CK] × 100

where CK refers to the DI value of negative control and T refers to the DI value of compound **1** and Polyoxin B.

### 4.6. Data Analysis

Analysis of variance (ANOVA) was conducted using SPSS Statistics 22.0 (IBM/SPSS, Chicago, IL, USA). Treatment means were separated by Tukey’s Honestly Significant Difference test (*p* = 0.05).

## 5. Conclusions

Three compounds were isolated and identified from the plant endophytic actinomycete strain R6, including one new and one known LOB. Unlike previous related studies [19,20,33,34,35,36,37,38], the LOBs isolated in this study originate from plant endophytic actinomycetes and exhibit certain inhibitory activities against both plant pathogenic bacteria and fungi. *In vitro* tests demonstrated that LOB H8 effectively inhibited three bacterial strains. Furthermore, *in vivo* tests confirmed the bioactivity of LOB H8 against *B. cinerea*. These results highlight the potential of lobophorins as promising lead compounds for the development of new, sustainable agents to control plant diseases. On this basis, further studies will be conducted to elucidate its mechanism of action, providing a theoretical foundation and basis for the development of new antifungal agents to prevent and treat gray mold disease and to enrich the variety of antifungal agents available.

## Figures and Tables

**Table 1:** The ^1^H (600 MHz) and ^13^C (150 MHz) NMR data of compounds **1** and **2** in CDCl_3_ (*δ*, ppm).

No.	1		No.	2	
	*δ*_H_, (mult, *J* in Hz)	*δ*_C_, mult		*δ*_H_, (mult, *J* in Hz)	*δ*_C_, mult
1	-	167.2 C	1	-	167.1 C
2	-	102.2 C	2	-	102.1 C
3	-	206.4 C	3	-	206.1 C
4	-	50.9 C	4	-	50.8 C
5	2.01 (t-like, 9.1)	42.7 CH	5	2.01 (t-like, 9.5)	43.2 CH
6	1.60 (m)	31.1 CH	6	1.62 (m)	31.2 CH
7	1.60 (overlap), 1.54 (m)	41.6 CH_2_	7	1.64 (m), 1.54 (m)	41.7 CH_2_
8	2.26 (m)	34.6 CH	8	2.27 (m)	34.0 CH
9	3.66 (dd, 10.5, 5.3)	76.1 CH	9	3.57 (m)	85.6 CH
10	2.07 (td, 10.0, 2.4)	39.1 C	10	2.18 (m)	38.3 CH
11	6.03 (br d, 10.0)	125.3 CH	11	5.74 (br d, 10.0)	124.7 CH
12	5.43 (m)	126.6 CH	12	5.45 (m)	127.3 CH
13	3.53 (m)	53.1 CH	13	3.56 (m)	52.9 CH
14	-	135.7 C	14	-	135.2 C
15	5.22 (d, 10.0)	122.9 CH	15	5.23 (d, 10.0)	123.2 CH
16	2.37 (overlap), 2.22 (m)	31.8 CH_2_	16	2.37 (overlap), 2.20 (m)	31.8 CH_2_
17	4.19 (br s)	72.9 CH	17	4.19 (br s)	72.9 CH
18	-	140.7 C	18	-	140.7 C
19	5.26 (d, 10.5)	118.2 CH	19	5.27 (d, 10.5)	118.2 CH
20	3.58 (br d, 10.5)	40.2 CH	20	3.58 (br d, 10.5)	40.2 CH
21	5.14 (br s)	120.6 CH	21	5.14 (br s)	120.5 CH
22	-	137.6 C	22	-	137.7 C
23	2.37 (overlap)	31.8 CH	23	2.37 (overlap)	31.8 CH
24	2.37 (m), 1.80 (m)	35.2 CH_2_	24	2.38 (m), 1.80 (m)	35.2 CH_2_
25	-	83.0 C	25	-	83.0 C
26	-	201.1 C	26	-	201.0 C
27	1.61 (s)	15.1 CH_3_	27	1.60 (s)	15.0 CH_3_
28	0.65 (d, 7.0)	22.2 CH_3_	28	0.65 (d, 7.0)	22.1 CH_3_
29	1.04 (d, 6.8)	12.9 CH_3_	29	1.08 (d, 6.8)	14.4 CH_3_
30	1.38 (s)	13.7 CH_3_	30	1.36 (s)	13.7 CH_3_
31	1.38 (s)	14.7 CH_3_	31	1.39 (s)	14.8 CH_3_
32	1.77 (s)	21.8 CH_3_	32	1.77 (s)	21.8 CH_3_
33	1.28 (d, 6.8)	20.1 CH_3_	33	1.29 (d, 6.8)	20.1 CH_3_
	-		1′	4.95 (d, 3.6)	99.6 CH
	-		2′	2.37 (m), 1.94 (dt, 14.6, 3.5)	34.9 CH_2_
	-		3′	3.99 (m)	67.1 CH
	-		4′	3.16 (br d, 7.0)	72.6 CH
	-		5′	3.83 (m)	65.3 CH
	-		6′	1.34 (d, 6.8)	17.7 CH_3_

**Table 2:** Antibacterial effect of the isolated compounds.

Compounds	Minimum Inhibitory Concentration (MIC, μg/mL)			
	*PsL*	*PsT*	*Cm*	*Ec*
Lobophorin H8 (**1**)	15.63	7.81	250	3.91
Lobophorin S (**2**)	62.5	15.63	500	62.5
Divergolide C (**3**)	250	125	500	31.25
Streptomycin	7.81	3.91	500	31.25

*PsL* = *Pseudomonas syringae* pv. *lachrymans; PsT* = *P. syringae* pv. tomato; *Cm* = *Clavibater michiganensis*; *Ec* = *Erwinia carotovora*.

**Table 3:** *In vitro* antifungal activity of the isolated compounds.

Compounds	Minimum Inhibitory Concentration (MIC, μg/mL)							
	*So*	*Ff*	*Bc*	*As*	*Pa*	*Pg*	*Fo*	*Cc*
Lobophorin H8 (**1**)	62.5	31.25	1.95	62.5	>500	500	62.5	500
Lobophorin S (**2**)	250	62.5	7.81	250	500	>500	250	500
Divergolide C (**3**)	250	31.25	15.63	125	62.5	500	62.5	125
Amphotericin B	500	125	125	500	62.5	>500	125	125
Carbendazim	500	62.5	31.25	125	15.63	31.25	15.63	>500
Polyoxin B	125	31.25	3.91	31.25	250	500	125	62.5

*So *=* Stemphylium solani; Ff *=* Fulvia fulva; Bc *=* Botrytis cinerea; As *=* Alternaria solani; Pa *=* Pythium aphanidermatum; Pg *=* Pyricularia grisea; Fo *=* Fusarium oxysporum; Cc *=* Corynespora cassiicola.*

**Table 4:** The control effect of lobophorin H8 (**1**) on tomato grey mold disease.

Treatment	Dilute Multiple (µg/mL)	Disease Index	Control Effect (%) ^a^
Lobophorin H8 (**1**)	0.49	38.00	35.22 ± 1.90 e
	0.98	28.67	51.16 ± 3.25 d
	1.95	12.63	78.51 ± 3.80 b
	3.91	11.33	80.73 ± 2.42 ab
	7.81	8.44	85.44 ± 3.29 a
Polyoxin B	1.95	17.23	70.70 ± 3.81 c
CK	-	58.63	-

^a^ Note: The different letters behind the numbers mean significant differences (*p* = 0.05). CK: negative control.

## Data Availability

The data are contained within the article and Supplementary Materials.

## Supplementary Materials

The following supporting information can be downloaded at: https://www.mdpi.com/article/10.3390/molecules30030586/s1, 16S rDNA sequence of *Streptomyces* sp. R6 isolated from *Azadirachta indica*; Figure S1: Phylogenetic tree of actinomycete R6; Figures S2–S9: HPLC, HRESIMS, ^1^H NMR, ^13^C NMR, HSQC, HMBC, ^1^H-^1^H COSY, and NOESY spectrum of lobophorin H8 (**1**); Figures S2 and S10–S16: HPLC, HRESIMS, ^1^H NMR, ^13^C NMR, HSQC, HMBC, ^1^H-^1^H COSY, and NOESY spectrum of lobophorin S (**2**); Figures S17–S20: HPLC, HRESIMS, ^1^H NMR, and ^13^C NMR spectrum of divergolide C (**3**); Figure S21: Inhibitory effects of lobophorin H8 (**1**) on *Botrytis cinerea*.
