# Supplementary material for: Antimicrobial Lobophorins from Endophytic Strain Streptomyces sp. R6 Obtained from Azadirachta indica
Source: Molecules. 2025 Jan 27;30(3):586. doi: 10.3390/molecules30030586 (PMC11820565; doi:10.3390/molecules30030586)
Supplement: Supplementary file 1 [file molecules-30-00586-s001.zip › molecules-3382924-supplementary.pdf]

**Supporting information**

# Antimicrobial Lobophorins from Endophytic Strain *Streptomyces* sp. R6 Obtained from *Azadirachta indica*

**16S rDNA sequence of *Streptomyces* sp. R6 isolated from *Azadirachta indica***

GCCAAGTGCGGTCTTACACATGCAGTCGAACGATGAACCACTTCGGTG
GGGATTAGTGGCGAACGGGTGAGTAACACGTGGGCAATCTGCCCTGCACT
CTGGGACAAGCCCTGGAAACGGGGTCTAATACCGGATATTGACCTTCACGG
GCATCTGTGAGGTTCGAAAGCTCCGGCGGTGCAGGATGAGCCCGCGGCCT
ATCAGCTTGTTGGTGAGGTAATGGCTCACCAAGGCGACGACGGGTAGCCG
GCCTGAGAGGGCGACCGGCCACACTGGGACTGAGACACGGCCCAGACTC
CTACGGGAGGCAGCAGTGGGGAATATTGCACAATGGGCGAAAGCCTGATG
CAGCGACGCCGCGTGAGGGATGACGGCCTTCGGGTTGTAAACCTCTTTCA
GCAGGGAAGAAGCGAAAGTGACGGTACCTGCAGAAGAAGCGCCGGCTAA
CTACGTGCCAGCAGCCGCGGTAATACGTAGGGCGCAAGCGTTGTCCGGAAT
TATTGGGCGTAAAGAGCTCGTAGGCGGCTTGTCACGTCGGTTGTGAAAGCC
CGGGGCTTAACCCCGGGTCTGCAGTCGATACGGGCAGGCTAGAGTTCGGTA
GGGGAGATCGGAATTCCTGGTGTAGCGGTGAAATGCGCAGATATCAGGAG
GAACACCGGTGGCGAAGGCGGATCTCTGGGCCGATACTGACGCTGAGGAG
CGAAAGCGTGGGGAGCGAACAGGATTAGATACCCTGGTAGTCCACGCCGT
AAACGGTGGGCACTAGGTGTGGGCAACATTCCACGTTGTCCGTGCCGCAG
CTAACGCATTAAGTGCCCCGCCTGGGGAGTACGGCCGCAAGGCTAAAACT
CAAAGGAATTGACGGGGGCCCGCACAAGCGGCGGAGCATGTGGCTTAATT
CGACGCAACGCGAAGAACCTTACCAAGGCTTGACATACACCGGAAACGGC
CAGAGATGGTCGCCCCCTTGTGGTCGGTGTACAGGTGGTGCATGGCTGTCG
TCAGCTCGTGTCGTGAGATGTTGGGTTAAGTCCCGCAACGAGCGCAACCCT
TGTCCCGTGTTGCCAGCAAGCCCCTTCGGGGGTGTTGGGGACTCACGGGA
GACCGCCGGGGTCAACTCGGAGGAAGGTGGGGACGACGTCAAGTCATCAT
GCCCCTTATGTCTTGGGCTGCACACGTGCTACAATGGCCGGTACAATGAGC
TGCGATACCGCAAGGTGGAGCGAATCTCAAAAAGCCGGTCTCAGTTCGGA
TTGGGGTCTGCAACTCGACCCCATGAAGTCGGAGTCGCTAGTAATCGCAGA
TCAGCATTGCTGCGGTGAATACGTTCCCGGGCCTTGTACACACCGCCCGTC

ACGTCACGAAAGTCGGTAACACCCGAAGCCGGTGGCCCAACCCCTTGTGG
GAGGGAGCTTCGAAGGTGACTAAACTT

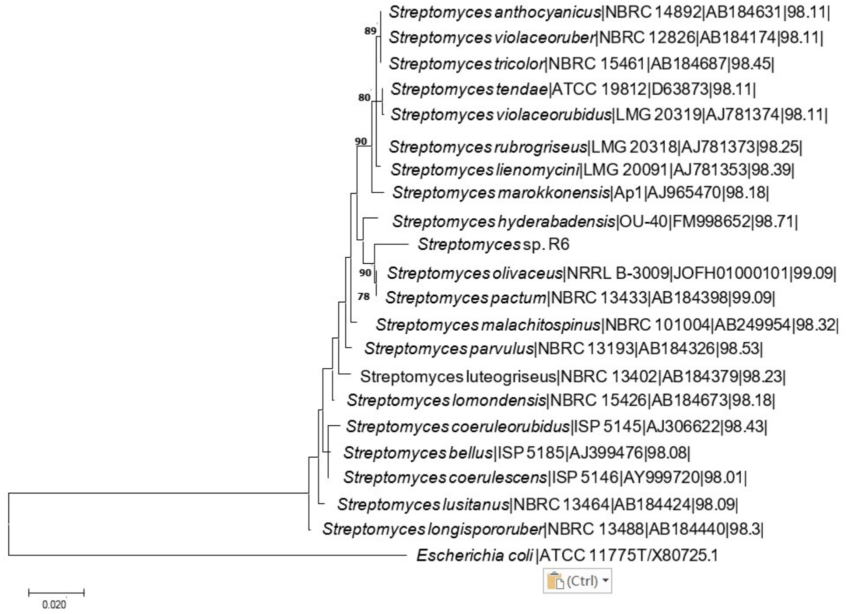


**Figure S1. Phylogenetic tree of Actinomycete *Streptomyces* sp. R6**

*The phylogenetic tree of strain R6 was constructed based on the 16S rRNA gene sequence using the maximum likelihood (ML) method. The Poisson correction model was employed with 1200 bootstrap replications, and bootstrap values greater than 70% are indicated at the branch nodes. The scale bar represents a nucleotide difference distance of 0.020 per unit.

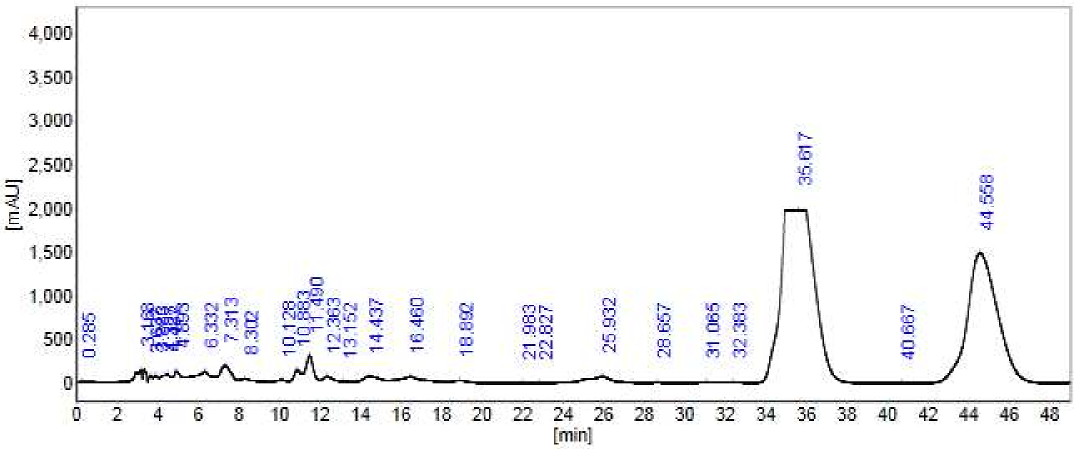


**Figure S2. The HPLC analysis of lobophorin H8 (1) and lobophorin S (2), $t_R$=35.62 for compound 1; $t_R$=44.56 for compound 2.**

## 1. Lobophorin H8 (1)

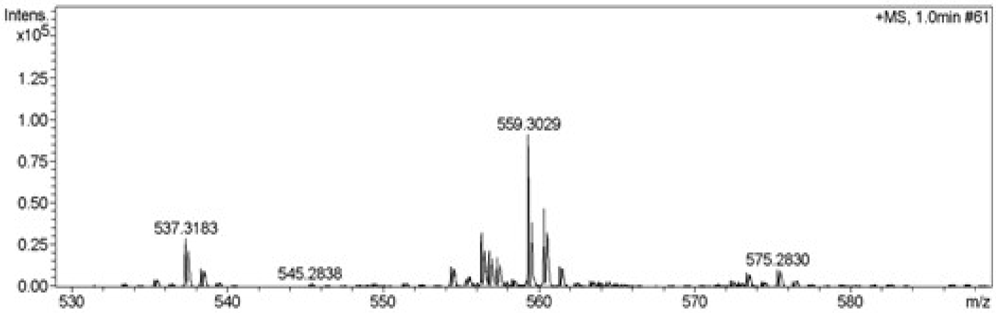


**Figure S3. HRESIMS spectrum of lobophorin H8 (1).**

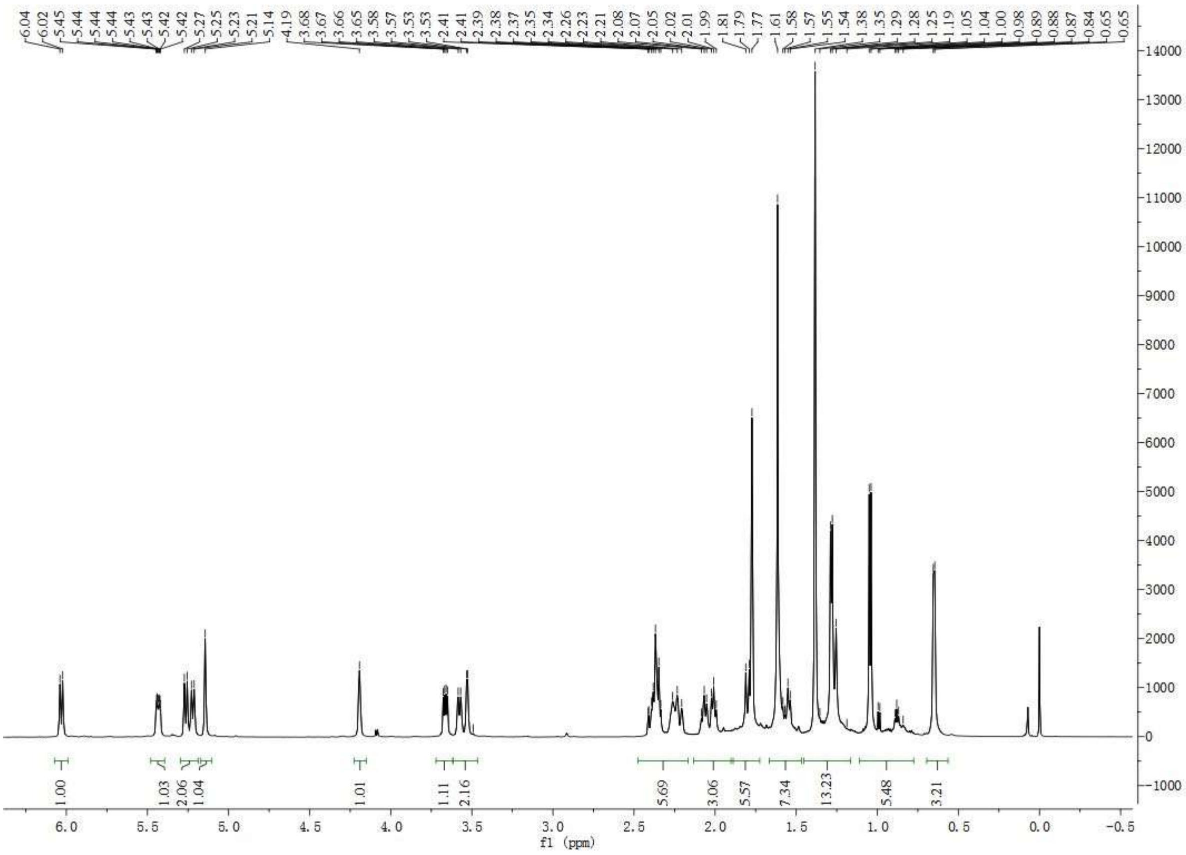


**Figure S4. $^{1}$H NMR spectrum of lobophorin H8 (1).**

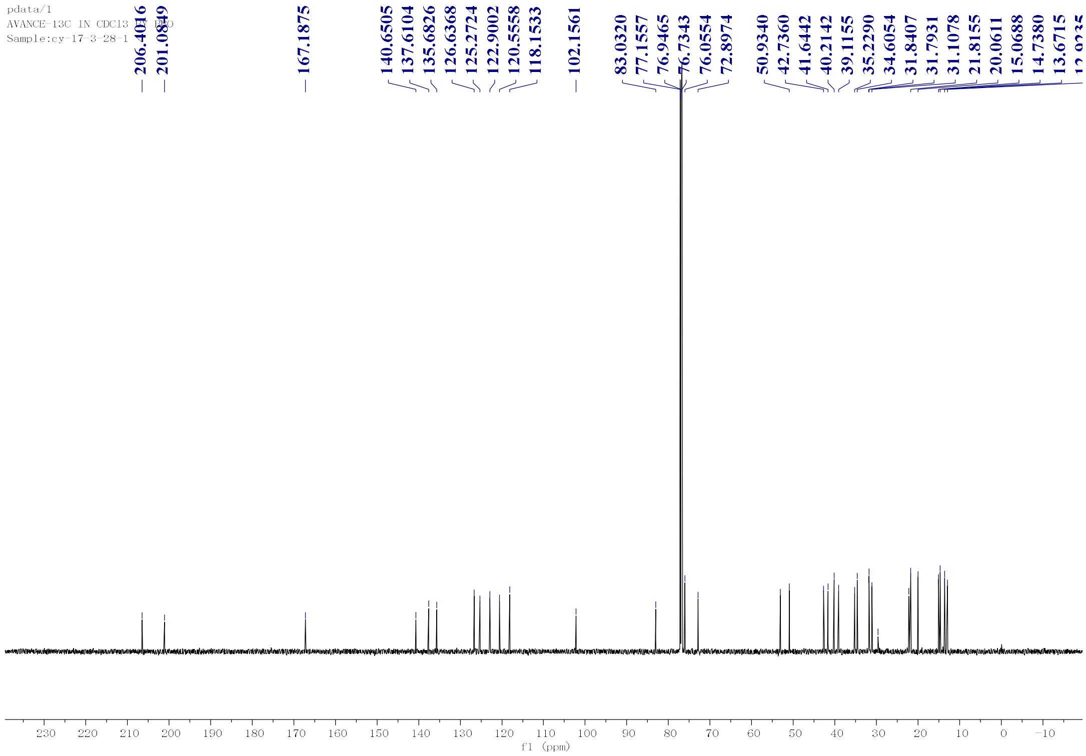


**Figure S5. $^{13}$C NMR spectrum of lobophorin H8 (1).**

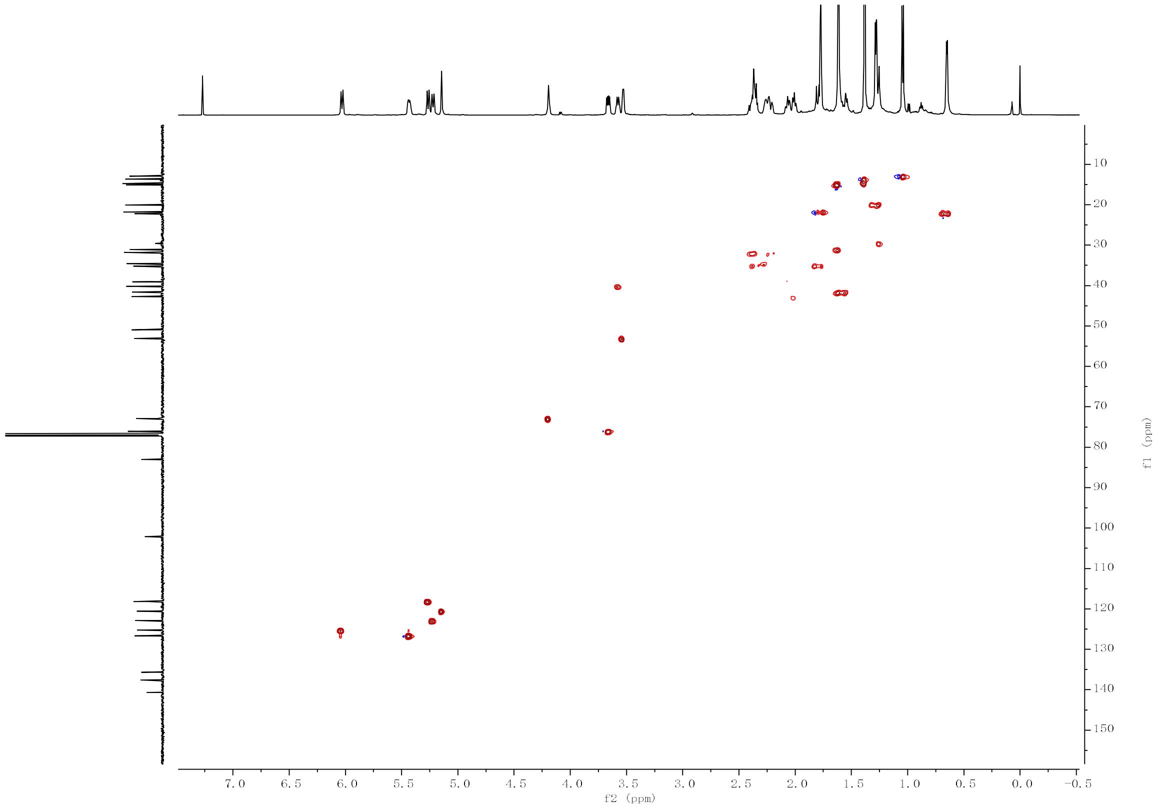


**Figure S6. HSQC spectrum of lobophorin H8 (1).**

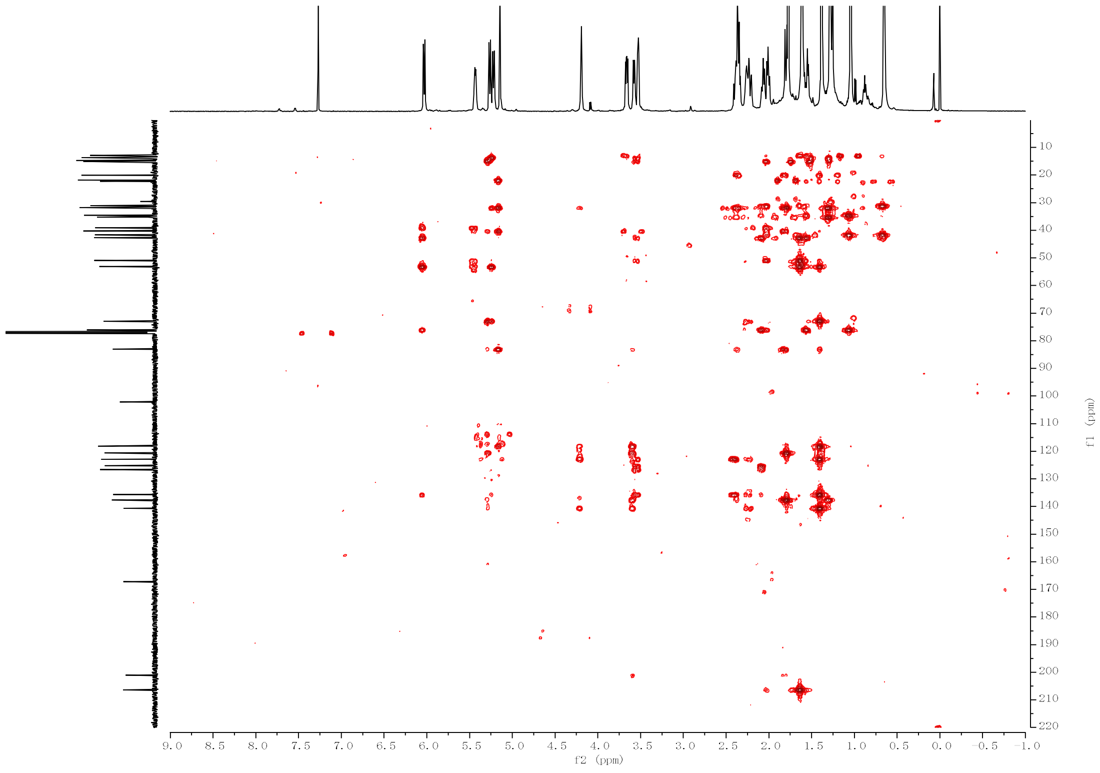


**Figure S7. HMBC spectrum of lobophorin H8 (1).**

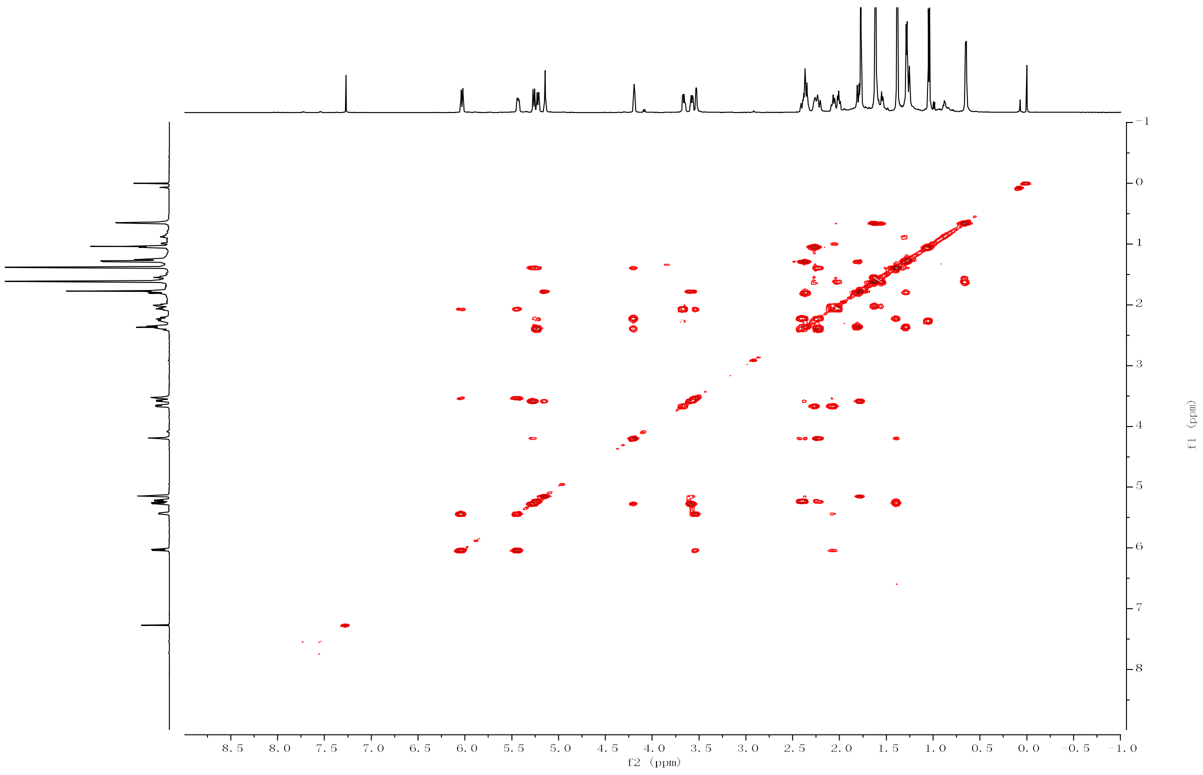


**Figure S8. $^{1}$H-$^{1}$H COSY spectrum of lobophorin H8 (1).**

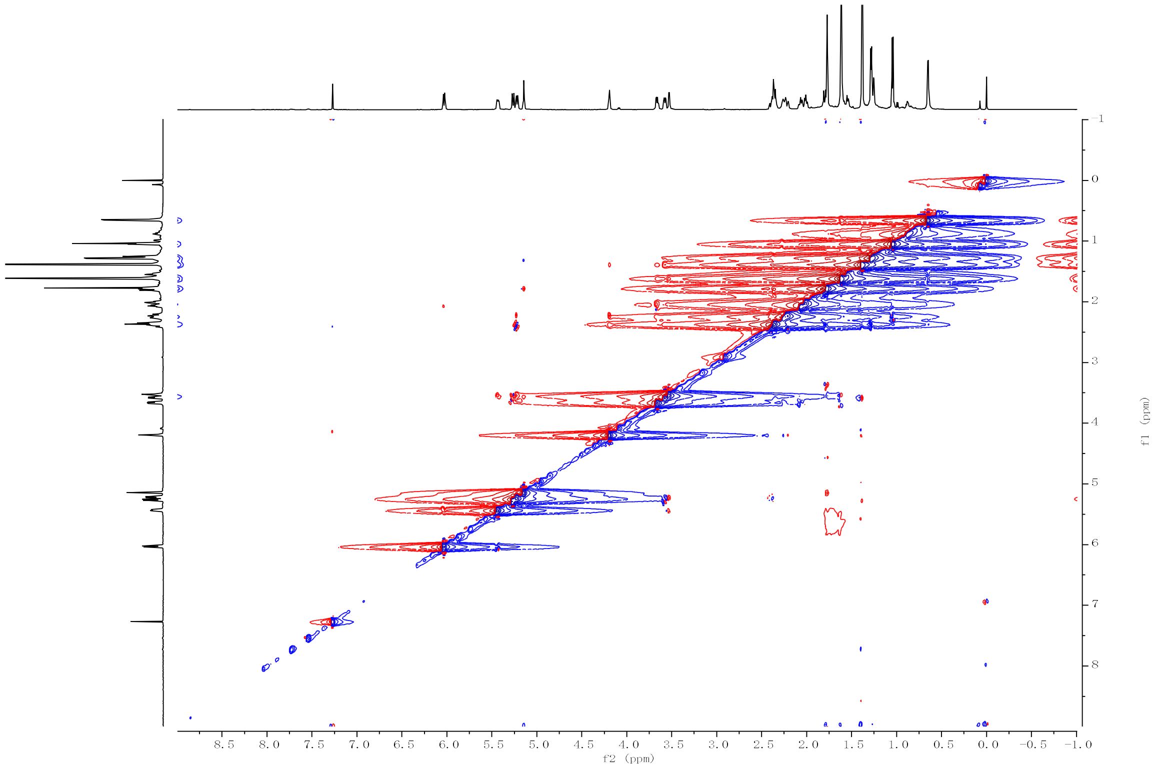


**Figure S9. NOESY spectrum of lobophorin H8 (1).**

## 2. lobophorin S (2)

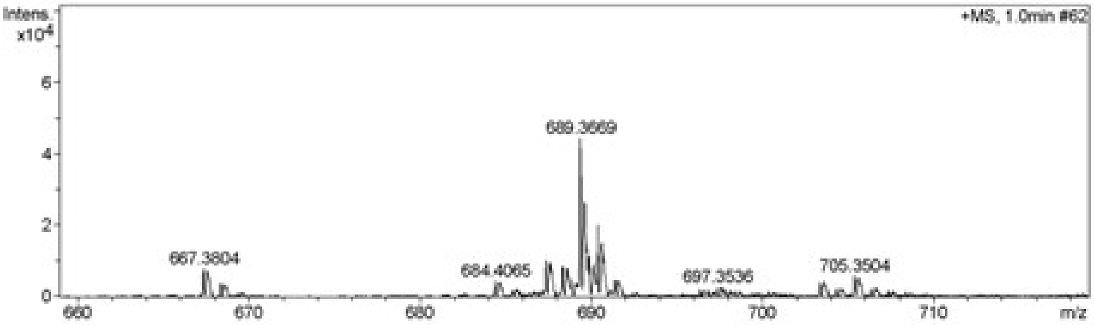


**Figure S10. HRESIMS spectrum of lobophorin S (2).**

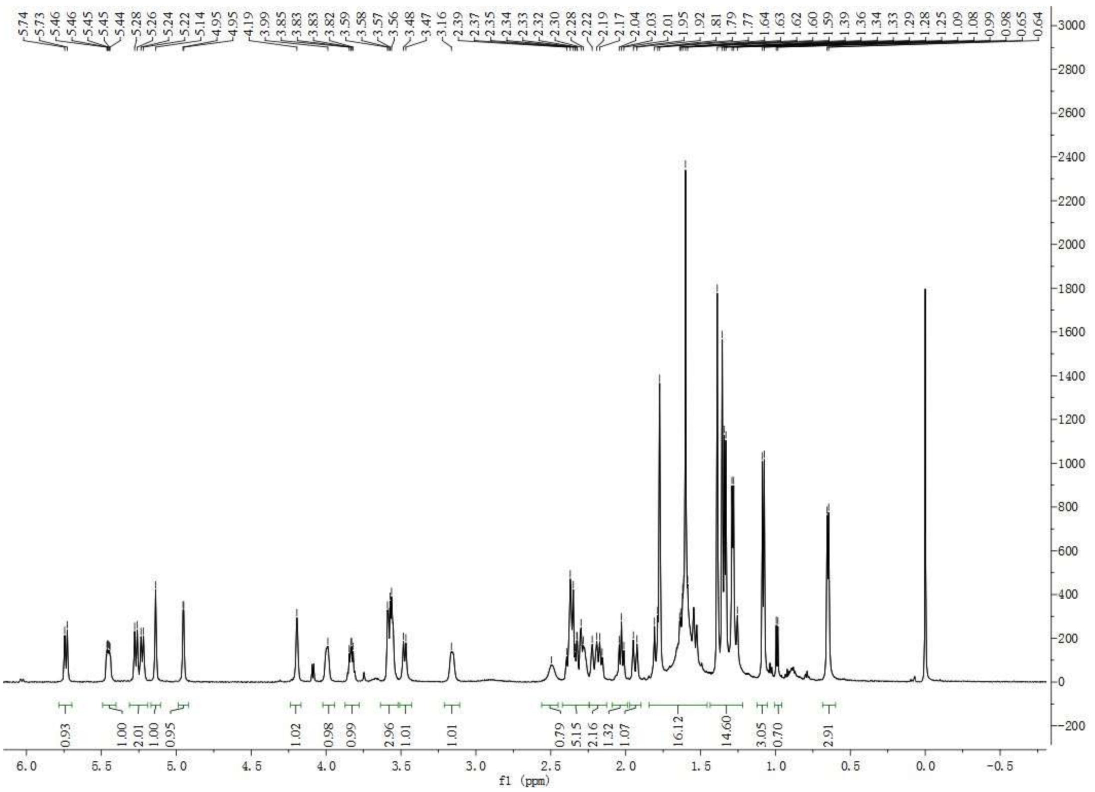

**Figure S11. $^{1}$H NMR spectrum of lobophorin S (2).**

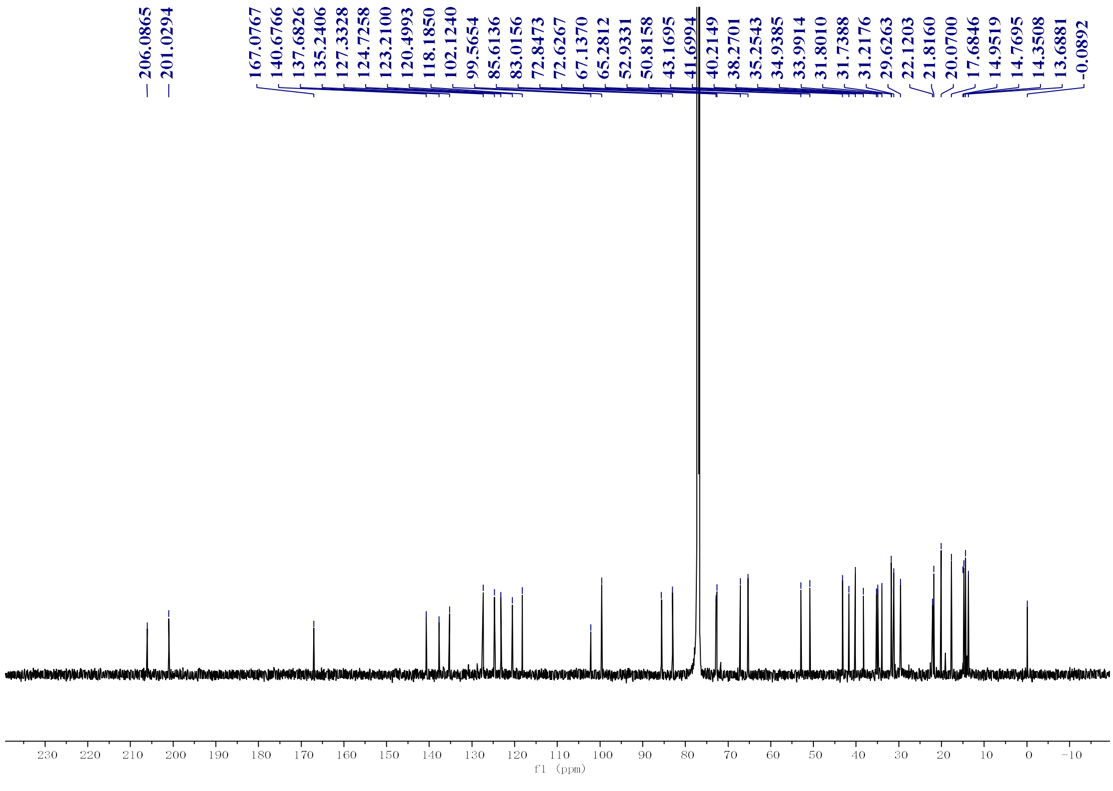

**Figure S12. $^{13}$C NMR spectrum of lobophorin S (2).**

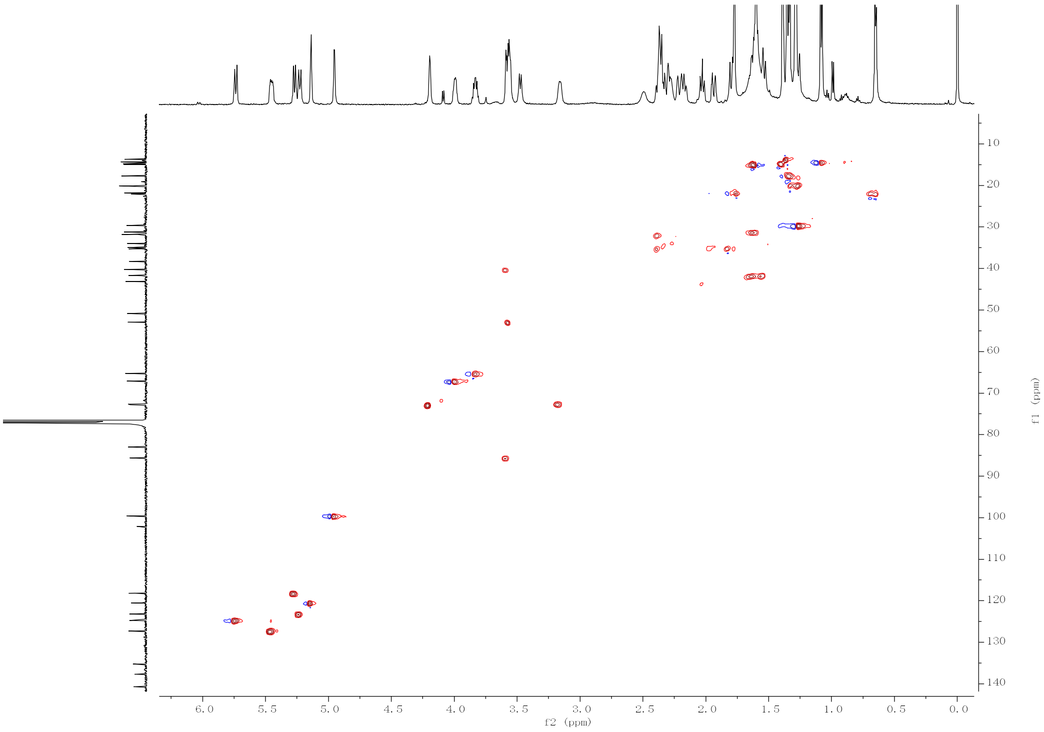

**Figure S13. HSQC spectrum of lobophorin S (2).**

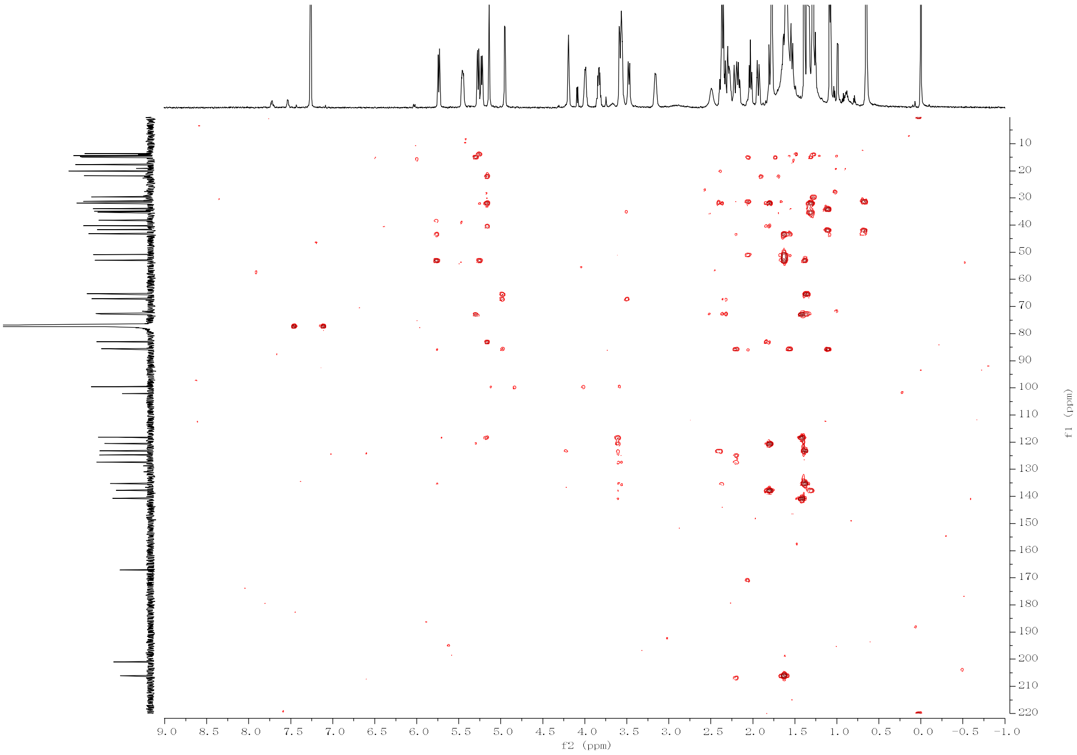

**Figure S14. HMBC spectrum of lobophorin S (2).**

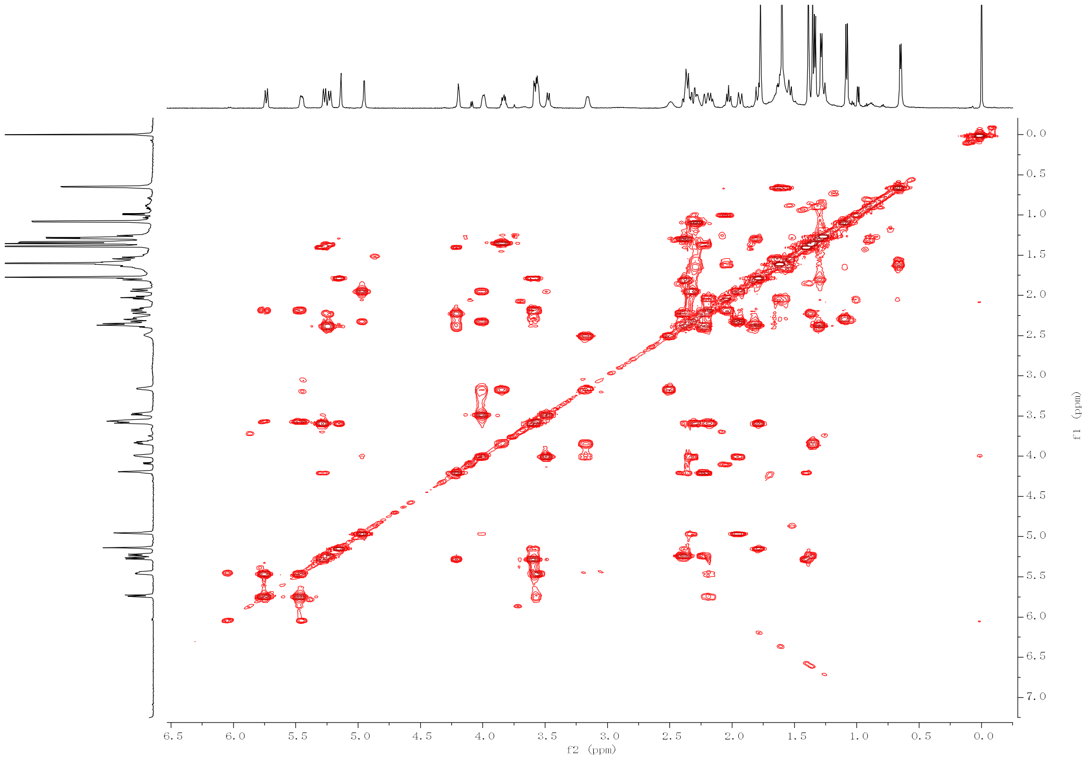


**Figure S15. $^{1}$H-$^{1}$H COSY spectrum of lobophorin S (2).**

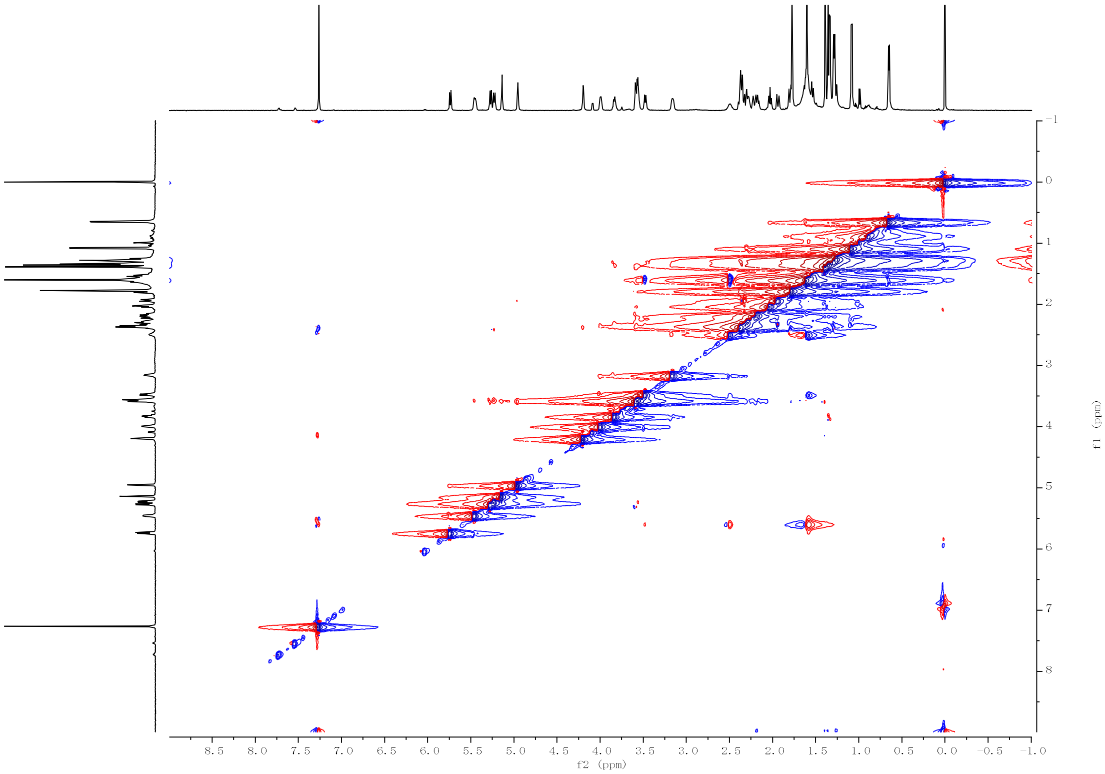


**Figure S16. NOESY spectrum of lobophorin S (2).**

## 3. Divergolide C

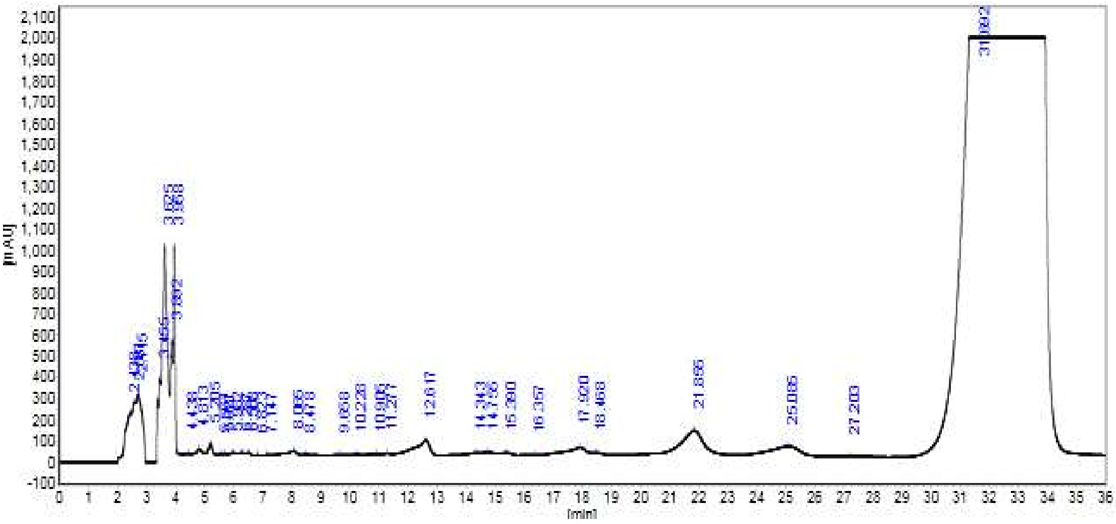

Figure S17. The HPLC analysis of divergolide C.

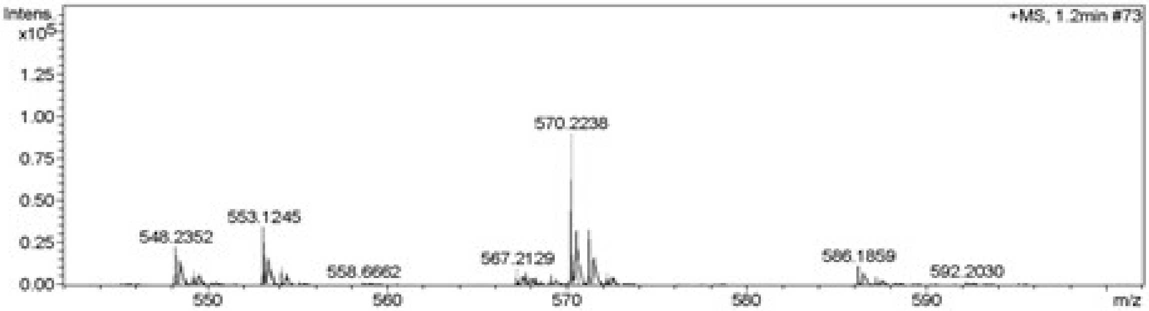


Figure S18. HRESIMS spectrum of divergolide C.

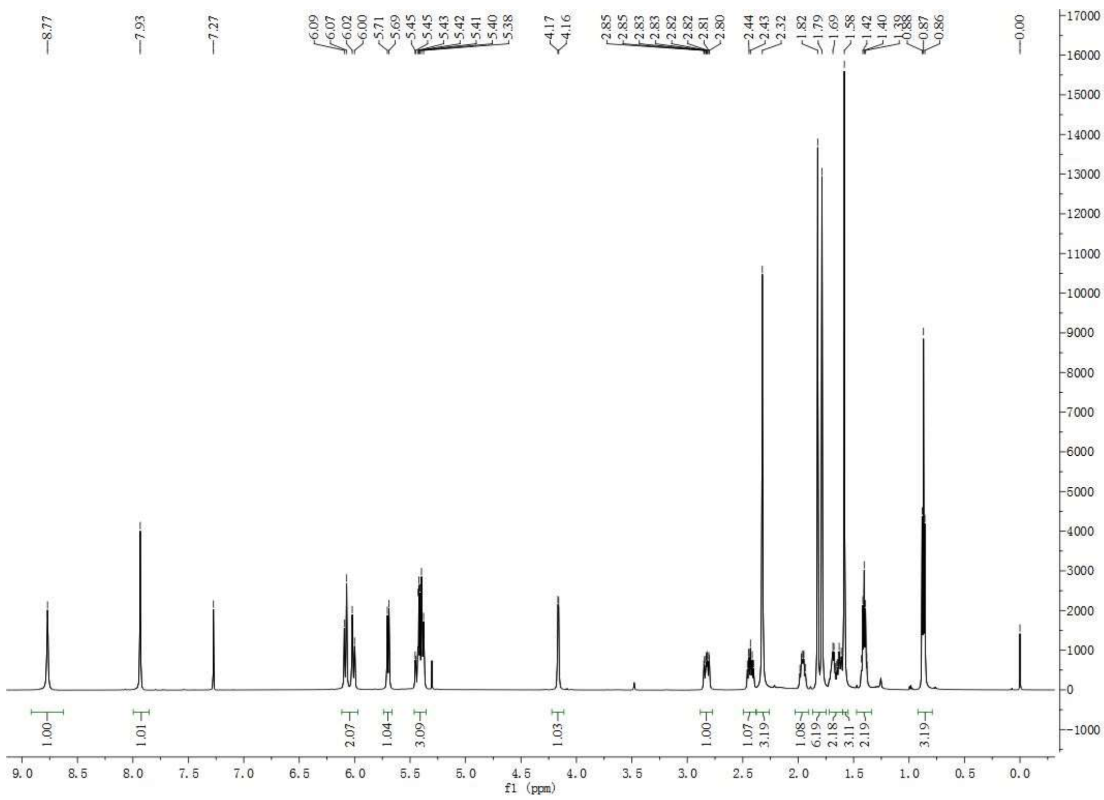


**Figure S19. $^{1}$H NMR spectrum of divergolide C.**

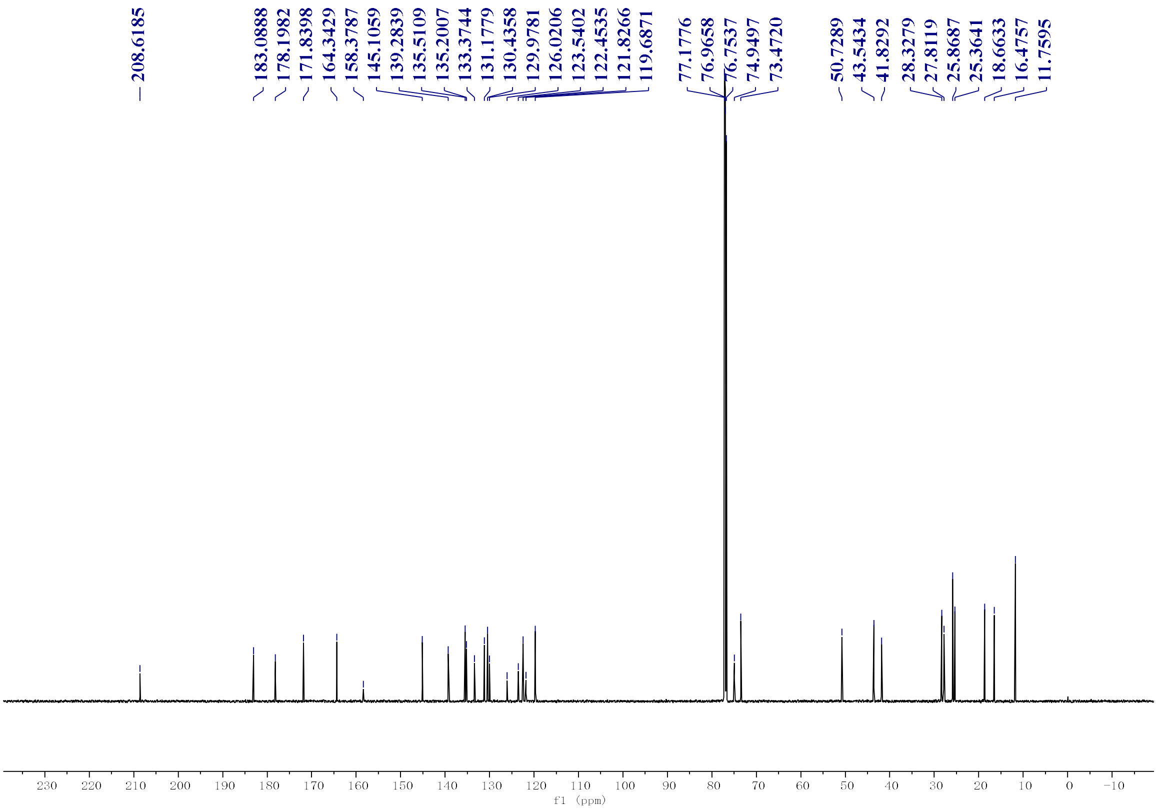


**Figure S20. $^{13}$C NMR spectrum of divergolide C.**

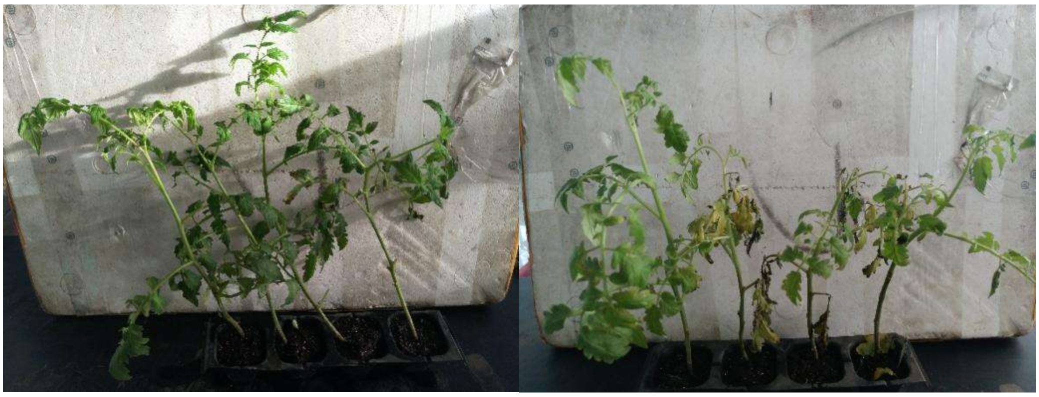

lobophorin H8 (1) against *Botrytis cinerea*

Negative control of *Botrytis cinerea* infection tomato

**Figure S21. Inhibitory effects of lobophorin H8 (1) on *Botrytis cinerea in vivo*.**
